# Targeting ferroptosis in cancer: molecular mechanisms and emerging therapeutic strategies

**DOI:** 10.1016/j.bbrep.2026.102825

**Published:** 2026-10-09

**Authors:** Mansoor Khaledi, Hasti Ahani, Faezeh Rezaei Tohid, Fatemeh Kazemi, Elham Amirkhani, Zeynab Daliri, Sahar Yadegari, Kosar Moarefvand, Behnam Poureslamfar, Fatemeh Sameni, Sajjad Yazdansetad

**Affiliations:** a Cellular and Molecular Research Center, Basic Health Sciences Institute, Shahrekord University of Medical Sciences, Shahrekord, Iran; b Department of Surgery, Razi Clinical Research Development Unit, Guilan University of Medical Sciences, Rasht, Iran; c Department of Molecular Cell Biology, Faculty of Animal Science, Sari Agricultural Sciences and Natural Resources University, Sari, Iran; d Medical Student, School of Medicine, Alborz University of Medical Sciences, Karaj, Iran; e Department of General Medicine, Faculty of Medicine, Kashan University of Medical Sciences, Kashan, Iran; f Department of Biology, Division of Cell and Developmental Biology, Faculty of Basic Sciences, Science and Research Branch, Islamic Azad University, Tehran, Iran; g Medical Laboratory Supervisor, Department of Medical Laboratory Sciences, Faculty of Medical Sciences, Islamic Azad University, Tehran, Iran; h I.M. Sechenov First Moscow State Medical University, Ministry of Health of the Russian Federation (Sechenov University), Moscow, Russia; i Department of Microbiology, Faculty of Medicine, Shahed University, Tehran, Iran; j Infectious Diseases and Tropical Medicine Research Center, Shahid Beheshti University of Medical Sciences, Tehran, Iran; k Department of Biology, Faculty of Basic Sciences, Imam Hossein Comprehensive University, Tehran, Iran

**Keywords:** Ferroptosis, Lipid peroxidation, Cancer therapy, Nanoparticles, GPX4

## Abstract

Ferroptosis, an iron-dependent form of regulated cell death characterized by the lethal accumulation of lipid peroxides, has emerged as a pivotal pathway in cancer biology and a promising therapeutic target. This review synthesizes the core molecular machinery governing ferroptosis, detailing the critical roles of iron metabolism through systems like transferrin/transferrin receptor 1 and ferritinophagy, the cystine/glutamate antiporter system (Xc^−^)/glutathione/GPX4 axis, and the recently discovered FSP1/CoQ10 pathway. We further explore the intricate interplay between lipid peroxidation, amino acid metabolism, and mitochondrial dysfunction, highlighting how their convergence dictates cellular susceptibility to ferroptotic death. The therapeutic potential of inducing ferroptosis in oncology is extensively reviewed, encompassing classic small-molecule inducers (e.g., erastin, RSL3, sorafenib) that target these key pathways. A significant focus is placed on the cutting-edge application of nanotechnology, discussing how various nanoparticles, including iron-based and organic systems, can potently induce ferroptosis through Fenton reactions, glutathione depletion, and targeted drug delivery, thereby overcoming traditional chemoresistance. We also introduce cold atmospheric plasma (CAP) as an emerging physical inducer of ferroptosis. Moreover, we examine the dynamic crosstalk between ferroptosis and the tumor immune microenvironment, particularly how CD8^+^ T cells and tumor-associated macrophages can modulate or respond to ferroptotic signals. Beyond summarizing the molecular basis of ferroptosis, this review critically integrates emerging advances in biomarker discovery, nanotechnology-enabled therapeutic strategies, and translational oncology to provide a clinically oriented perspective on ferroptosis-based cancer therapy. By highlighting biomarker-guided patient stratification, mechanisms of therapeutic resistance, and the principal challenges limiting clinical implementation, including tumor heterogeneity, off-target toxicity, pharmacokinetic constraints, and drug delivery barriers, this review provides a comprehensive framework for the rational development of precision oncology strategies targeting ferroptosis and identifies key priorities for future clinical translation.

## Highlights

• Reviews ferroptosis mechanisms: iron metabolism, lipid peroxidation, and ROS
• Details emerging ferroptosis inducers, including nanoparticles and small molecules
• Explores ferroptosis in cancer therapy and immunotherapy
• Discusses combination strategies to overcome ferroptosis resistance
• Outlines future directions for biomarkers and clinical translation

## 1 Introduction

Carcinogenesis is characterized by the evasion of apoptosis regulation to achieve unlimited proliferation and immortality [1]. Recent advances in genomics and proteomic technologies, along with rapid progress in interpreting gene expression datasets, have enabled researchers to identify genes involved in ferroptosis [2].

Ferroptosis is a form of regulated cell death linked to iron homeostasis and uncontrolled lipid peroxidation. It is distinct from other types of cell death and plays a critical role in most cancers [3]. Ferroptosis has recently gained attention as a targetable vulnerability in many cancer types [4].

In 2012, Dixon proposed ferroptosis as a new mode of cell death [5]. Compared to autophagy and apoptosis, ferroptosis is characterized by cytoplasmic alterations, such as the shrinking or absence of mitochondrial cristae, the rupturing of the mitochondrial outer membrane, and the contraction of the mitochondrial membrane [6]. This type of cell death is caused by the disruption of plasma membrane integrity resulting from excessive lipid peroxidation [7]. Ferroptosis is an adaptive process that can aid in the elimination of cancer cells [8] by removing cells deprived of nutrients, water, or damaged by infection or environmental stress. It also helps prevent tumorigenesis [9].

Ferroptosis caused by cysteine deficiency is significantly reduced with the activation of autophagy protein 13 (ATG-13), in addition to the degradation and downregulation of autophagy protein 3 (ATG-3) [10].

Mitochondria can influence ferroptosis through their roles in cellular metabolism, reactive oxygen species (ROS) production, iron handling, and the regulation of redox homeostasis. However, their contribution is context dependent and should not be interpreted as indicating that mitochondria are universally required for ferroptotic cell death. Experimental studies have shown that mitochondrial metabolism can enhance ferroptosis under specific metabolic conditions, particularly during cysteine deprivation, when glutaminolysis, tricarboxylic acid cycle activity, and mitochondrial ROS generation can contribute to lipid-peroxidative stress. In contrast, ferroptosis triggered by direct inhibition of GPX4 can occur in the absence of normal mitochondrial respiratory activity in some experimental systems. Mitochondria therefore function primarily as context-dependent modulators of ferroptosis susceptibility rather than as an obligatory execution mechanism. In addition, mitochondrial antioxidant pathways, including the dihydroorotate dehydrogenase (DHODH)-mediated CoQH_2_ defense system, can suppress lipid peroxidation under specific conditions, further emphasizing the dual role of mitochondria in either promoting or limiting ferroptosis [11,12].

Ferroptosis, a type of reactive oxygen species (ROS)-related cell death, results in free iron overload (FIO), ROS production, lipid peroxidation, and ultimately membrane damage [5]. Morphological features of ferroptosis include the disruption of plasma membrane integrity, mild chromosomal atrophy, swelling of cytoplasm and organelles, and mitochondrial changes such as elevated membrane density and outer membrane rupture [13]. FIO results from decreased iron metabolism and the accumulation of intracellular or extracellular iron. This can lead to excessive ROS production through the Fenton process, causing oxidative DNA damage, lipid peroxidation, and membrane damage [6,14,15]. In addition, the activation of iron-containing enzymes, such as lipoxygenases, by FIO can increase the rate of membrane lipid peroxidation [10]. Enzymes that contribute to lipid oxidation and ferroptotic cell death include lipoxygenase and long-chain acyl-CoA synthetase (ACSL4). ACSL4 is essential for the synthesis of polyunsaturated fatty acid-containing phospholipids (PUFA-PLs), while lipoxygenases catalyze the oxidation of PUFA-PLs, leading to the production of lipid hydroperoxides and active aldehydes such as 4-hydroxynonenal and malondialdehyde [16]. Cytochrome P450 oxidoreductase (POR) also drives lipid peroxidation [17].

Although numerous review articles have summarized the molecular mechanisms of ferroptosis, most have focused predominantly on individual signaling pathways or specific therapeutic modalities [18]. The present review provides a broader translational perspective by integrating the fundamental molecular machinery governing ferroptosis, including iron metabolism, lipid peroxidation, the System Xc^−^/GSH/GPX4 axis, and the FSP1/CoQ10 pathway, with emerging determinants of therapeutic response, including tumor heterogeneity, the tumor immune microenvironment, and metabolic plasticity. In addition, we critically evaluate recently developed ferroptosis-inducing strategies such as nanoparticle-based delivery systems and cold atmospheric plasma, emphasizing their translational potential as well as the challenges that currently limit clinical implementation. Unlike previous reviews, we further discuss clinically relevant biomarkers for predicting ferroptosis sensitivity, compare the developmental stages of available therapeutic approaches, and highlight major barriers to clinical translation, including off-target toxicity, pharmacokinetic limitations, tumor heterogeneity, and resistance mechanisms [19]. By integrating these molecular, technological, and clinical aspects within a single framework, this review provides an updated and clinically oriented perspective on ferroptosis-based cancer therapy while identifying key priorities for future precision oncology.

## 2 Key players and pathways

It is important to recognize that the molecular regulation of ferroptosis is highly interconnected rather than being organized into isolated signaling pathways [20]. Many ferroptosis regulators exhibit pleiotropic functions by simultaneously modulating iron metabolism, antioxidant defense systems, lipid remodeling, mitochondrial metabolism, and stress-responsive signaling networks [21]. Consequently, several regulatory proteins and pharmacological agents may appear in more than one mechanistic category throughout this review. Such classifications are intended to emphasize their predominant mechanisms of action while acknowledging that ferroptosis is governed by extensive molecular crosstalk and dynamic feedback regulation rather than by independent linear pathways [22]. Central to ferroptotic cell death are iron, lipids, and reactive oxygen species. The accumulation of ROS is driven by the dysregulation of multiple pathways, including the classic Fenton reaction, glutathione peroxidase 4 (GPX4) inactivation, and lipid peroxidation [17]. Lipid peroxides derived from polyunsaturated fatty acids catalyze the propagation of the ferroptotic process, resulting in cellular damage [23].

### 2.1 The role of iron in ferroptosis

Iron is an essential nutrient for cellular metabolism and plays a dual role in ferroptosis. First, it generates ROS through the Fenton reaction when reacting with hydrogen peroxide [24]. Second, iron is a vital component of GPX4, which degrades lipid hydroperoxides and preserves cellular membrane integrity [25]. Maintaining a delicate balance in iron levels and utilization is pivotal in regulating ferroptosis [26].

### 2.2 Involvement of lipid peroxidation

Lipid peroxidation is a hallmark of ferroptosis. It involves the uncontrolled peroxidation of polyunsaturated fatty acids within cellular membranes. This damages membrane structure, disrupts fluidity and permeability, and compromises essential functions such as ion transport and signaling. Ultimately, it leads to cellular demise [27].

### 2.3 Regulatory machinery of ferroptosis

Numerous regulators and signaling pathways fine-tune ferroptotic cell death. The Keap1-Nrf2 pathway, p53, and autophagy machinery exert pro- or anti-ferroptotic effects, often acting together to maintain cellular homeostasis [28]. Understanding these networks provides insights into potential therapeutic targets for diseases with dysregulated ferroptosis [29]. While ferroptosis is involved in various non-cancer pathologies (summarized in Supplementary Table S1), this review focuses on its implications in oncology.

### 2.4 Glutathione peroxidase 4 (GPX4)

Recent evidence further indicates that GPX4 functions as a central integrative node linking cellular antioxidant capacity with lipid metabolic homeostasis and ferroptosis sensitivity. Nevertheless, GPX4-independent ferroptosis suppression pathways, including the FSP1–CoQ10 axis, GCH1–BH4 signaling, and DHODH-mediated mitochondrial antioxidant defense, demonstrate that ferroptosis susceptibility cannot be explained solely by GPX4 activity. Instead, multiple complementary antioxidant systems cooperate to determine cellular responses to oxidative stress, thereby contributing to tumor-specific heterogeneity and differential therapeutic sensitivity [30].

### 2.5 Ferroptosis suppressor protein 1 (FSP1)

The discovery of FSP1 has fundamentally reshaped the current understanding of ferroptosis regulation by demonstrating that inhibition of GPX4 alone is not universally sufficient to induce ferroptotic cell death [31]. Instead, the relative contributions of GPX4-dependent and GPX4-independent antioxidant systems vary considerably among different tumor types, emphasizing the importance of biomarker-guided therapeutic strategies for identifying patients most likely to benefit from ferroptosis-targeted therapies [30].

### 2.6 Techniques and experimental approaches

Researchers employ various methods to study ferroptosis in vitro and in vivo [17]. These include small-molecule inhibitors, genetic manipulation of ferroptosis genes, and lipid peroxidation assays [32]. These tools provide clues on induction and regulation mechanisms [1].

### 2.7 Potential therapeutic strategies

Understanding ferroptosis mechanisms offers opportunities for interventions [33]. Pharmacological agents that selectively induce or inhibit ferroptosis promise avenues for cancer treatment, neuroprotection, or oxidative stress conditions [34].

#### 2.7.1 Molecular crosstalk among ferroptosis regulatory networks

Although ferroptosis is frequently described through discrete molecular pathways, accumulating evidence indicates that these regulatory systems operate as an integrated signaling network [35]. Alterations in iron metabolism directly influence lipid peroxidation, while glutathione metabolism, GPX4 activity, FSP1-dependent antioxidant defense, mitochondrial metabolism, and stress-responsive transcription factors collectively determine ferroptosis sensitivity [36]. Moreover, metabolic reprogramming within the tumor microenvironment further modulates these interactions through changes in nutrient availability, hypoxia, inflammatory signaling, and immune cell activity [37]. Consequently, therapeutic strategies targeting multiple regulatory nodes simultaneously may achieve greater antitumor efficacy than approaches directed toward a single molecular target, particularly in heterogeneous tumors exhibiting adaptive resistance mechanisms.

## 3 Regulatory mechanisms of ferroptosis

The key molecular regulators introduced in Section 2 function through interconnected metabolic and signaling pathways that collectively determine ferroptosis susceptibility. The following sections discuss these regulatory mechanisms in greater mechanistic detail, emphasizing their roles in cancer biology and therapeutic intervention [38].

### 3.1 Metabolic iron

The Fenton reaction enhances ROS production by peroxidizing polyunsaturated fatty acids (PUFAs) with Fe^2+^. Thus, Fe^2+^ accumulation drives ferroptosis. Intracellular Fe^2+^ levels are regulated by complex events, including iron metabolism, which influences ferroptosis (Fig. 1).

**Fig. 1:**
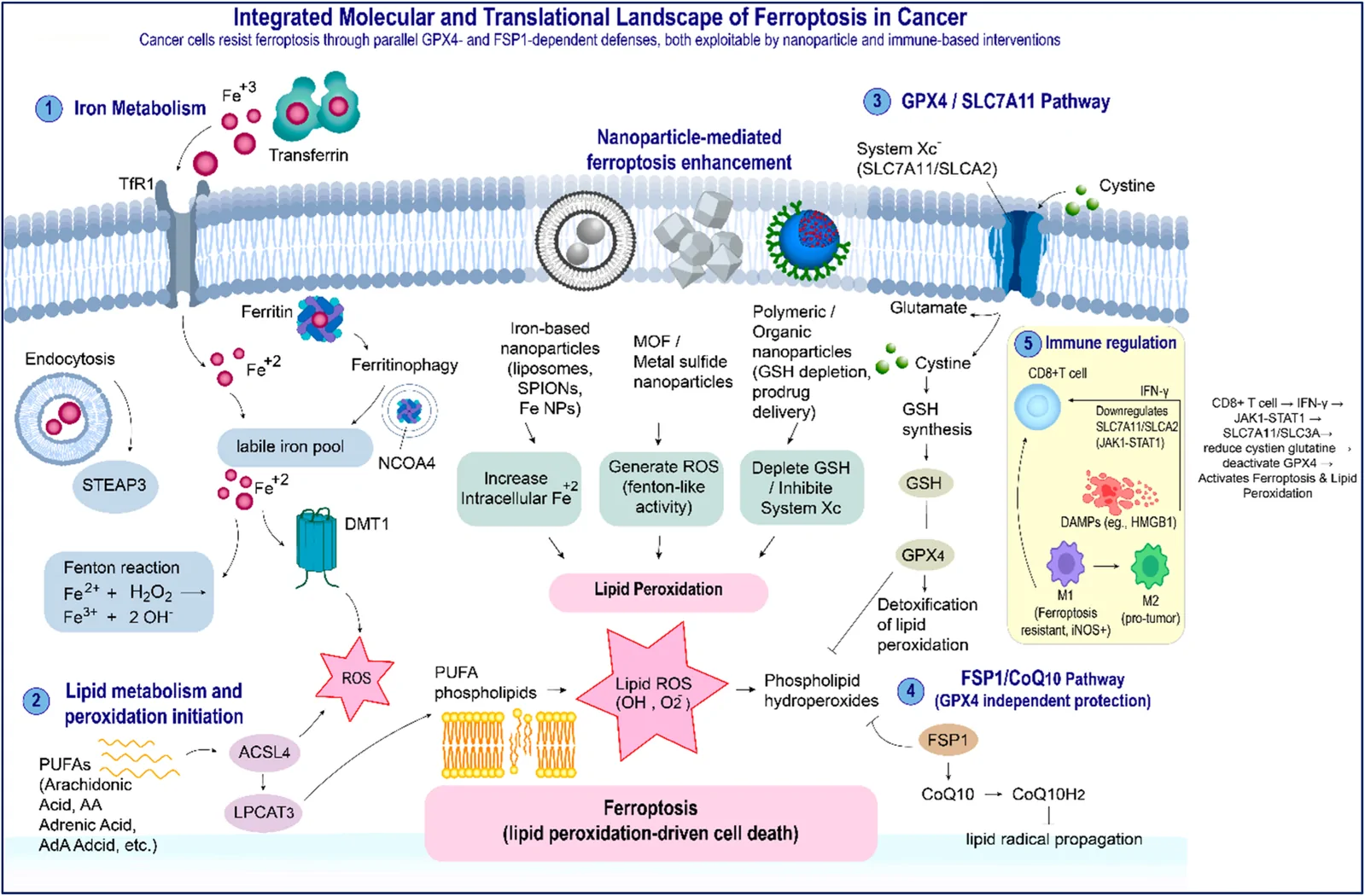
**Integrated molecular and translational landscape of ferroptosis in cancer.** Ferroptosis is an iron-dependent form of regulated cell death driven by the accumulation of lipid peroxides and insufficient antioxidant protection. The schematic integrates the principal molecular pathways that determine ferroptosis susceptibility and illustrates how iron metabolism, lipid remodeling, antioxidant defenses, immune regulation, and nanoparticle-based therapeutic strategies converge to promote or suppress ferroptotic cancer-cell death. **(1) Iron metabolism:** Transferrin-bound Fe^3+^ is internalized through transferrin receptor 1 (TFR1) and reduced to Fe^2+^, contributing to the labile iron pool. Ferritinophagy mediated by nuclear receptor coactivator 4 (NCOA4) releases stored iron and increases the availability of redox-active Fe^2+^. Iron accumulation promotes Fenton chemistry and reactive oxygen species (ROS) generation, thereby facilitating lipid peroxidation. **(2) Lipid metabolism and peroxidation:** Polyunsaturated fatty acids (PUFAs), including arachidonic acid, adrenic acid, and docosahexaenoic acid, are incorporated into membrane phospholipids through lipid-remodeling enzymes such as ACSL4 and LPCAT3. PUFA-containing phospholipids are susceptible to oxidation, generating lipid ROS and phospholipid hydroperoxides (PL-OOH), which contribute directly to ferroptotic membrane damage. **(3) The SLC7A11/GSH/GPX4 axis:** The cystine/glutamate antiporter system Xc^−^, composed of the light chain SLC7A11 and the heavy chain SLC3A2, imports cystine in exchange for glutamate and supports cysteine availability and GSH synthesis. GPX4 uses GSH to reduce phospholipid hydroperoxides and thereby protects cells from ferroptotic damage. Disruption of cystine uptake, GSH synthesis, or GPX4 activity compromises this antioxidant defense and promotes lipid-peroxide accumulation. **(4) The FSP1/CoQ_10_ pathway:** Ferroptosis suppressor protein 1 (FSP1) provides a GPX4-independent defense mechanism by maintaining the reduced CoQ_10_ pool, thereby suppressing lipid-radical propagation and limiting ferroptosis. **(5) Immune regulation and the tumor microenvironment:** Immune-cell interactions can modulate ferroptosis sensitivity. CD8^+^ T-cell-derived interferon-γ (IFN-γ) can suppress SLC7A11/System Xc^−^ activity in cancer cells, reducing cystine availability and weakening antioxidant defenses. Macrophage polarization and tumor-associated macrophage (TAM) states can further influence ferroptosis and the immunological characteristics of the tumor microenvironment (TME). **Nanoparticle-mediated ferroptosis enhancement:** Iron-based nanoparticles, metal-organic framework (MOF)/metal-sulfide nanoparticles, and polymeric or organic nanoparticles can enhance ferroptosis through complementary mechanisms, including increasing intracellular iron, promoting ROS generation and Fenton chemistry, depleting GSH or inhibiting System Xc^−^, and facilitating targeted therapeutic delivery. Collectively, these pathways illustrate the convergence of iron-driven oxidative stress, PUFA-dependent lipid peroxidation, and insufficient antioxidant protection, while immune modulation and nanoparticle-based interventions provide opportunities to enhance ferroptotic cancer-cell death and remodel the tumor microenvironment. Abbreviations: AA, arachidonic acid; ACSL4, acyl-CoA synthetase long-chain family member 4; AdA, adrenic acid; CoQ10, coenzyme Q10; DHA, docosahexaenoic acid; DMT1, divalent metal transporter 1; FSP1, ferroptosis suppressor protein 1; GSH, glutathione; GPX4, glutathione peroxidase 4; IFN-γ, interferon-γ; LPCAT3, lysophosphatidylcholine acyltransferase 3; MOF, metal-organic framework; NCOA4, nuclear receptor coactivator 4; PL-OOH, phospholipid hydroperoxides; PUFA, polyunsaturated fatty acid; ROS, reactive oxygen species; SLC3A2, solute carrier family 3 member 2; SLC7A11, solute carrier family 7 member 11; STEAP3, six-transmembrane epithelial antigen of prostate 3; TAMs, tumor-associated macrophages; TFR1, transferrin receptor 1; TME, tumor microenvironment; Xc-, cystine/glutamate antiporter system.

Ferroptosis depends on iron transport. Transferrin (TF), lactotransferrin (LTF), and iron-saturated lactoferrin (holo-LF) sensitize cells to ferroptosis [39]. Ferroptosis is marked by transferrin receptor 1 (TfR1), which imports iron [40]. Ceruloplasmin (CP)-assisted iron export by ferroportin (FPN) prevents ferroptosis [41]. The membrane glycoprotein prominin-2 exports ferritin via multivesicular bodies and exosomes, preventing cellular ferroptosis [42]. FPN is the primary iron efflux source.

Ferritin's FTH1 and FTL subunits store intracellular Fe^2+^. FTH1 depletion promotes ferroptosis [43]. NCOA4-mediated ferritinophagy is a selective autophagic process in which ferritin is delivered to lysosomes for degradation, thereby releasing stored iron and increasing the availability of redox-active Fe^2+^. By expanding the labile iron pool, this process can enhance susceptibility to ferroptosis. However, lysosomal involvement in ferroptosis should be interpreted primarily in the context of specific processes such as ferritinophagy and iron mobilization rather than as evidence that lysosomes constitute a universal execution mechanism for ferroptotic cell death [44,45].

Unbound Fe^2+^ in mitochondria increases erastin-induced ferroptosis, but mitochondrial ferritin (FtMt) sequesters iron and resists it. Heme breakdown by heme oxygenase-1 (HO-1) releases Fe^2+^; HO-1 regulates ferroptosis [44].

The iron-sulfur cluster (ISC) complex maintains iron homeostasis and redox balance. ISC deficiency induces ferroptosis. Cysteine desulfurase NFS1 and frataxin produce ISCs; NFS1 generates sulfur from cysteines, reducing Fe^2+^ and blocking ferroptosis [46]. Frataxin transfers Fe^2+^ to cysteine, activating NFS1; its deficiency disrupts homeostasis and induces ferroptosis [47]. NEET proteins transport iron regulatory proteins (IRPs) to ISCs. IRPs and IREs elevate iron by increasing TfR1 and suppressing ferritin/FPN, inducing ferroptosis [48]. Inhibiting ISC-IRP binding via NEET targets blocks ferroptosis.

### 3.2 GPX4: the central guardian of lipid peroxidation

Glutathione peroxidase 4 (GPX4) is a phospholipid hydroperoxidase that directly reduces lipid hydroperoxides to lipid alcohols, thereby terminating the chain reaction of lipid peroxidation. Unlike other GPX family members, GPX4 is unique in its ability to interact with complex lipid membranes [25]. Its active site contains a selenocysteine residue, which is critical for catalytic activity and is incorporated via a specific SECIS element in its 3′-UTR [49]. Genetic or pharmacological inactivation of GPX4 is sufficient to trigger ferroptosis, even in the presence of other antioxidant systems [50]. Small-molecule inducers such as RSL3 and ML162 covalently bind to the selenocysteine of GPX4, irreversibly inhibiting its activity. FIN56, another inducer, promotes GPX4 degradation [51]. The expression of GPX4 is transcriptionally regulated by TFAP2c and Sp1, and its activity depends on selenium bioavailability; selenium supplementation upregulates GPX4 and protects against ferroptosis. In cancer, high GPX4 expression is frequently observed and correlates with poor prognosis and resistance to chemotherapy [52]. Notably, cancer stem cells often rely on GPX4 to escape lipid peroxidation, making them particularly susceptible to GPX4 inhibitors. Thus, targeting GPX4 remains one of the most promising strategies for inducing ferroptosis in oncology [53].

### 3.3 ROS metabolism

Superoxide dismutase (SOD) converts O_2_^−^ to H_2_O_2_, while catalase (CAT), peroxiredoxins (PRDXs), and glutathione peroxidases (GPXs) convert H_2_O_2_ to water (Fig. 1). Cofactors include thioredoxin (TRX), reduced glutathione (GSH), glutathione-S-transferases (GSTs), PRDX, and GPX. GSH removes oxidative stress-related molecules [41,54]. NADPH acts as an electron donor to regenerate GSH and TRX. NADPH, used by immune cells, is synthesized from NADP^+^ in mammalian cells [55,56]. Production is limited by phosphatases like cytosolic MESH1 and mitochondrial Nocturnin but boosted by NAD^+^ kinase (NADK) [57,58]. Antioxidant pathways interconnect for stress responses, but ROS scavenging is often linear. SOD can increase HO levels without GPX [57,59]. NADPH supports anabolic activities and NOXs that generate ROS [60,61]; homeostasis is essential [62,63].

Mitophagy removes damaged ROS-producing mitochondria [64], though it can boost ROS under certain conditions [65].

### 3.4 Amino acid and glutathione metabolism

Specialized transporters facilitate amino acid membrane transport. Solute carrier family 3 member 2 (SLC3A2) and solute carrier family 7 member 11 (SLC7A11) form the Xc^−^ heterodimeric system [66]. Cystine entry converts to cysteine for GSH synthesis. Targeting Xc^−^ induces ferroptosis [67]. Erastin, an early inducer, inhibits Xc^−^ by binding voltage-dependent anion channels (VDACs) in mitochondria, triggering ER stress. Sulfasalazine inhibits Xc^−^ and induces ferroptosis [68]. Sorafenib induces ferroptosis via Xc^−^ inhibition [69]. OTUB1 stabilizes SLC7A11; its deletion increases sensitivity [70]. ATF3 inhibits SLC7A11 transcription [71]. BAP1 reduces SLC7A11 by deubiquitinating H2A [72].

GSH, the main antioxidant, donates electrons to GPX4 to reduce lipid peroxides. Synthesis depends on glutamate, cysteine, and glycine via glutamate-cysteine ligase and GSH synthetase [73]. Buthionine sulfoximine (BSO) inhibits synthesis and induces ferroptosis [74]. Acetaminophen depletes GSH, causing ferroptosis. High phosphoenolpyruvate carboxykinase in CD8^+^ T cells maintains GSH and reduces ROS [75]. Overexpressing ABC transporters increases GSH flux and ferroptosis sensitivity [76]. These support targeting glutamate transport and GSH for cysteine cancer treatment [77]. Notably, the SLC7A11-GSH-GPX4 axis is frequently dysregulated in cancers, and its status predicts sensitivity to ferroptosis inducers [78].

### 3.5 Lipid metabolism

Lipid peroxidation is central to ferroptosis. Lethal LPO is a key feature. Organisms continuously produce ROS as aerobic byproducts, including lipid peroxides (ROOH), hydroxyl radicals (HO•), H_2_O_2_, and superoxide (O_2_^−^) [79]. ROS promotes proliferation, genetic instability, degeneration, and resistance in cancer [80].

Tumor cells express high antioxidants to maintain redox balance; imbalance causes death [81,82]. Enzymes like LOXs, NOXs, and COXs increase ROS [82]. Ferroptosis from LPO can be treated with lipophilic antioxidants [81]. LPO generation is regulated in ferroptosis. Characteristic mitochondrial morphological alterations are frequently observed during ferroptosis, but these changes should not be interpreted as evidence that mitochondria are universally required for ferroptotic cell death. Mitochondrial metabolism can promote ferroptosis in specific settings by supporting ROS production and metabolic pathways that increase cellular susceptibility to lipid peroxidation, particularly during cysteine deprivation. Conversely, direct GPX4 inhibition can induce ferroptosis in experimental systems with limited or altered mitochondrial function, indicating that mitochondrial activity is not an obligatory requirement for all ferroptotic responses. Thus, mitochondrial involvement is best understood as context dependent and closely linked to the metabolic state of the cell [11,12].

ACSL4 oxidizes AA and AdA to LPO; 15-LOX and LPCAT3 produce it. Iron catalyzes OH from H_2_O_2_, promoting PUFA chain reactions [81,83]. LOXs require Fe^2+^ as cofactor [7].

LPO has initiation, propagation, and termination phases. Initiation: ROS/RNS/RLS abstract H from allylic carbons in PUFAs, forming L•. Propagation: O_2_ + L• → LOO•, which abstracts H to form LOOH and new L•. Termination: Radicals react with each other or antioxidants like vitamin E/GSH, forming non-radicals [84].

Ferrous iron transforms LOOH to LO•, reacting with PUFAs for chain reactions. RSL3-induced ferroptosis overwhelms GPX4, accumulating LOOH that breaks to MDA/4-HNE, causing toxicity and signaling [68].

### 3.6 Association between lipid peroxidation, amino acid metabolism, and mitochondrial dysfunction

Ferroptosis results from the convergence of lipid susceptibility, iron-dependent oxidative stress, and inadequate antioxidant defense. Amino acid metabolism is particularly important because cysteine availability regulates glutathione synthesis and, consequently, GPX4-dependent detoxification of phospholipid hydroperoxides. Inhibition of cystine uptake or depletion of intracellular cysteine can therefore reduce glutathione availability and increase susceptibility to lipid-peroxidative damage [85].

Mitochondria can modify this response through context-dependent metabolic mechanisms. During cysteine deprivation, glutaminolysis and tricarboxylic acid cycle activity may enhance mitochondrial metabolism and ROS production, thereby contributing to ferroptosis sensitivity. However, mitochondrial ROS should not be regarded as the sole or universal source of the lipid-peroxidative stress responsible for ferroptosis. Lipid peroxidation can also proceed through iron-dependent and enzymatic mechanisms involving cellular membranes independently of a dominant mitochondrial contribution [86].

Mitochondria also contain protective mechanisms that can counteract ferroptosis. In particular, mitochondrial DHODH contributes to the reduction of CoQ to its antioxidant form and can suppress lipid peroxidation within mitochondrial membranes, providing a defense pathway that complements GPX4-dependent antioxidant activity. The relative contribution of mitochondrial metabolism to ferroptosis therefore depends on the initiating stimulus, metabolic state, antioxidant capacity, and cellular context. Accordingly, mitochondria should be considered dynamic regulators of ferroptosis susceptibility rather than uniformly pro-ferroptotic organelles [11,12].

## 4 Ferroptosis as a cancer therapy

The therapeutic literature on ferroptosis spans multiple levels of evidence, ranging from mechanistic cell-culture studies to animal experiments and, for a limited number of agents, human clinical investigation. These evidence levels should not be considered equivalent. Most ferroptosis-targeted approaches remain experimental, and promising activity in preclinical models does not establish clinical efficacy or safety. Throughout this section, therapeutic strategies are therefore discussed with attention to their current developmental stage and level of supporting evidence.

### 4.1 Small molecules inducers of ferroptosis

A growing number of small molecules can induce or sensitize cancer cells to ferroptosis by perturbing iron homeostasis, cystine uptake, glutathione metabolism, GPX4 activity, CoQ-dependent antioxidant defenses, or other pathways associated with lipid-peroxide accumulation. However, these agents differ substantially in their molecular selectivity and may influence multiple cellular processes. Accordingly, the compounds discussed below are classified according to their predominant reported mechanisms of action rather than as universally specific ferroptosis inducers. Representative agents and their principal molecular targets are summarized in Table 1.

**Table 1:** Molecular targets and predominant mechanisms of established and emerging ferroptosis-inducing agents.

Regulator/Agent	Principal target/pathway	Predominant mechanism	Evidence category	References
Erastin	System Xc^-^	Cystine uptake inhibition → GSH depletion	Well-established	[87]
Sulfasalazine	System Xc^-^	Cystine uptake inhibition	Well-established	[88]
BSO	GCL	Inhibits GSH synthesis	Well-established	[89]
RSL3	GPX4-associated antioxidant defense	Disrupts lipid peroxide detoxification	Well-established*	[90]
ML162	GPX4-associated antioxidant defense	Ferroptosis induction; mechanistic complexity reported	Well-established*	[91]
ML210/DPI10	GPX4-associated pathway	Cellularly activated ferroptosis inducer	Well-established	[92]
FIN56	GPX4/SQS–CoQ10	GPX4 degradation and CoQ-associated defense disruption	Well-established	[93]
Brequinar	DHODH	Inhibits mitochondrial ferroptosis defense	Emerging/established pathway	[94]
MMRi62	Ferritin/NCOA4-associated pathways	Promotes ROS and ferritin-related degradation	Emerging	[95]
NPD4928	FSP1	Inhibits FSP1-mediated defense	Emerging	[96]
Dexamethasone	GR–DPEP1–GSH	Sensitizes to ferroptosis through GSH depletion	Context-dependent	[97]
Statins	HMGCR/mevalonate pathway	Alters CoQ-associated metabolism	Context-dependent	[98]
Sorafenib	System Xc^−^-associated pathways	Reported ferroptosis-associated activity; controversial	Context-dependent	[99]
t-BuOOH	Oxidative stress	Experimental lipid oxidative stress inducer	Context-dependent	[100]

**Note:** Ferroptosis is regulated by highly interconnected molecular networks. Accordingly, several agents may influence more than one pathway. Each regulator is classified according to its predominant ferroptosis-related mechanism and current level of mechanistic evidence. “Well-established” indicates a reproducibly characterized role in ferroptosis regulation; “emerging” indicates a more recently described mechanism requiring further validation; and “context-dependent” indicates that the ferroptosis-related effect varies according to cell type, metabolic state, experimental conditions, or tumor context. Nanoparticle-based and physical ferroptosis-inducing strategies are summarized separately in Table 4.

**Table 2:** Molecular targets and predominant mechanisms of ferroptosis-suppressing regulators.

Regulator/Agent	Principal target/pathway	Anti-ferroptotic mechanism	Evidence category	References
DFO	Labile iron pool	Iron chelation	Well-established	[101]
DFP	Labile iron pool	Iron chelation	Well-established	[102]
DFX	Labile iron pool	Iron chelation	Well-established	[103]
CPX	Labile iron pool	Iron chelation	Well-established	[104]
Ferrostatin-1	Lipid radical propagation	Radical-trapping antioxidant	Well-established	[105]
Liproxstatin-1	Lipid radical propagation	Radical-trapping antioxidant	Well-established	[106]
α-Tocopherol	Lipid radical propagation	Lipophilic antioxidant	Well-established	[107]
Selenium-associated TFAP2C/SP1 pathway	GPX4/selenoproteins	Enhances protective selenoprotein expression	Emerging/context-dependent	[108]
β-ME	Cystine/cysteine availability	Experimental rescue of system Xc^−^ inhibition	Experimental	[109]
CDDO	HSP90/CMA	Stabilizes GPX4 in specific contexts	Context-dependent	[110]
Prominin-2	Ferritin/MVB/exosomes	Promotes ferritin-associated iron export	Emerging	[111]
miR-522	ALOX15	Reduces lipid ROS generation	Context-dependent	[112]
OTUB1	SLC7A11	Stabilizes SLC7A11	Emerging	[113]
LKB1–AMPK	ACC1/fatty-acid biosynthesis	Reduces lipid substrate availability	Context-dependent	[114]
iNOS/NO signaling	NO/lipid radical chemistry	Modulates ferroptosis depending on context	Context-dependent	[115]
NFE2L1	Proteasome homeostasis	Maintains adaptive proteasomal defense	Emerging	[116]

**Note:** Anti-ferroptotic regulation is mediated by multiple interconnected mechanisms, including iron sequestration, inhibition of lipid peroxidation, maintenance of the GSH/GPX4 axis, modulation of iron export, and proteostatic or metabolic adaptation. Regulators are classified according to their predominant mechanism and current level of supporting evidence. “Well-established” indicates reproducible anti-ferroptotic activity; “emerging” indicates a recently characterized or incompletely validated mechanism; and “context-dependent” indicates that the effect varies according to cellular, metabolic, or disease context.

In 2003, erastin, a small-molecule iron metabolism stimulator, was identified as a genotype-selective anticancer agent. It targets VDACs [81]. Loss of VDAC2/3 causes erastin resistance. Erastin binds VDAC2, producing lipid ROS. RSL5 binds VDAC3 similarly [117]. DIDS, a mitochondrial anion channel inhibitor, blocks VDACs [118,119], sensitizing to radiation [120]. Iron levels matter (Fig. 1). DMT1 relates to iron homeostasis [121]. Temozolomide induces ferroptosis via DMT1 in glioblastoma [122].

MMRi62 targets lipid metabolism. Sorafenib induces ferroptosis with ACSL4 in liver cancer [123], affecting lipid ROS. t-BuOOH affects lipid ROS, causing DNA damage and mitochondrial dysfunction, reversed by cardiolipin inhibitors [79]. It induces apoptosis via cell contacts [82].

#### 4.1.1 Small molecules targeting the GSH/GPX4 axis

GSH synthesis/oxidation links are targetable (Fig. 1). RSL3 targets GPX4's nucleophilic sites [117], like ML162, DPI7, DPI10 [124]. FIN56 degrades GPX4 [83]. BSO inhibits GCL, lowering GSH [125].

Erastin multitargets: inhibits VDACs, GSH, and GPX4. It reduces SLC7A5/SLC3A2 activity, affecting cysteine uptake [126]. Erastin lowers GSH and degrades GPX4 [110]. Analogs (MEII, PE, AE) enhance GSH depletion [74]. Sulfasalazine inhibits Xc^−^ [127]. Sorafenib impairs upstream [128]. NDP4928 potentiates ferroptosis via FSP1 [129].

#### 4.1.2 Drug inducers targeting the FSP1/CoQ pathway

NDP4928 weakly cytotoxic alone but enhances RSL3/BSO effects by binding FSP1 [129]. FIN56 binds squalene synthase and inhibits CoQ [130]. Statins inhibit HMGCR, lowering CoQ and inducing ferroptosis [131,132] (Fig. 1).

#### 4.1.3 Small molecules targeting other pathways

Brequinar blocks DHODH, inducing ferroptosis [133]. Dexamethasone upregulates DPEP1, depleting GSH [97]. Ferroptosis-modulating agents include both natural products and synthetic compounds. Artemisinin derivatives and other natural products have been reported to promote iron-dependent oxidative cell death in selected experimental cancer models, whereas synthetic compounds such as erastin and RSL3 have been widely used as experimental probes of ferroptosis. Nevertheless, the biological effects of these agents may depend on cell type, concentration, exposure duration, metabolic state, and the activity of compensatory antioxidant pathways. Therefore, evidence for ferroptosis should be interpreted within the experimental context and supported by appropriate mechanistic validation rather than inferred solely from the identity of the inducing agent [134].

#### 4.1.4 Cold atmospheric plasma as an emerging ferroptosis inducer

Cold atmospheric plasma (CAP) is an ionized gas operating at near-ambient temperature that generates a cocktail of reactive oxygen and nitrogen species (ROS/RNS), including •OH, O_2_•^-^, H_2_O_2_, NO•, and ONOO^−^ [135]. Recent studies have shown that CAP can potently induce ferroptosis in various cancer cell lines (e.g., glioblastoma, melanoma, pancreatic cancer) [136,137]. The mechanism involves CAP-derived ROS directly depleting intracellular glutathione (GSH) and inactivating GPX4, thereby allowing lipid peroxidation to proceed unchecked [138]. CAP also disrupts iron homeostasis by upregulating transferrin receptor 1 (TfR1) and downregulating ferritin heavy chain (FTH1), leading to increased labile iron pools and enhanced Fenton chemistry. Importantly, CAP treatment synergizes with conventional ferroptosis inducers (erastin, RSL3) and with nanoparticle-based delivery systems [139]. Because CAP can be applied directly to tumors via handheld devices [140], it offers a spatially targeted, non-invasive physical modality to trigger ferroptosis in difficult-to-treat or drug-resistant cancers [141]. Ongoing research aims to optimize CAP parameters (voltage, gas composition, exposure time) for clinical translation.

### 4.2 Specificity and limitations in the identification of ferroptosis induced by therapeutic agents

Although numerous pharmacological agents are widely used as ferroptosis inducers in experimental systems, their effects should not automatically be interpreted as evidence of a highly specific ferroptotic mechanism. Several compounds influence multiple molecular pathways, and excessive oxidative stress, mitochondrial injury, metabolic disruption, or membrane damage may coexist with apoptosis, necrosis, autophagy-associated processes, or other forms of regulated cell death. Consequently, increased ROS generation or lipid-peroxidation products alone are insufficient to establish ferroptosis as the predominant mechanism of cell death. Experimental attribution of ferroptosis should ideally be supported by convergent evidence, including the accumulation of lipid peroxidation, appropriate modulation of established ferroptosis regulators, rescue by validated ferroptosis inhibitors or radical-trapping antioxidants, and, where feasible, complementary genetic approaches. The interpretation should also consider whether the observed phenotype can be distinguished from alternative cell-death pathways. Accordingly, compounds discussed in this review should generally be regarded as ferroptosis-inducing or ferroptosis-sensitizing agents within defined biological contexts rather than universally specific inducers [142].

The therapeutic evidence supporting ferroptosis-targeted strategies remains highly heterogeneous. Many commonly used ferroptosis inducers and nanoplatforms have demonstrated activity primarily in cell culture or animal models, whereas clinical evidence remains limited. In addition, several established anticancer drugs have been reported to modulate ferroptosis experimentally but were not developed or approved as ferroptosis-targeted therapies [143]. Accordingly, evidence of ferroptosis induction in preclinical models should be clearly distinguished from clinical efficacy, and regulatory approval for a conventional anticancer indication should not be interpreted as clinical validation of ferroptosis-targeted therapy [144]. The developmental status of representative therapeutic strategies is summarized in Table 3.

**Table 3:** Current developmental status of ferroptosis-based therapeutic strategies in cancer.

Developmental Stage	Therapeutic Strategy/Inducer	Representative Cancer Type(s)	Primary Ferroptosis Mechanism	Current Development Status	References
FDA-approved	Sorafenib	Hepatocellular carcinoma, metastatic renal cell carcinoma, lung cancer	Inhibits System Xc^−^, promotes lipid ROS accumulation and ferroptosis	FDA-approved anticancer drug with reported ferroptosis-inducing activity	[145]
Clinical trials	Statins (HMG-CoA inhibitors)	Mesenchymal cancers	Mevalonate pathway inhibition, indirect GPX4 suppression	Investigated clinically; antitumor activity reported	[146]
Animal studies	Cyst(e)inase	HLRCC, CLL, prostate cancer	Cysteine depletion, ferroptosis induction	Demonstrated efficacy and acceptable safety in animal models	[147]
Animal studies	AlbADCA	Breast cancer	GPX4 inhibition, elimination of M2-TAMs	Suppressed tumor growth in vivo	[148]
Animal studies	RT-MPs	Cisplatin-resistant tumors	Macrophage repolarization, immunogenic ferroptosis	Enhanced antitumor immunity in animal models	[149]
Animal studies	Photosens (PS-PDT)	Experimental tumor models	PDT-mediated ROS generation and ferroptosis	Demonstrated ferroptosis induction and immune modulation	[150]
Animal studies	Oxygen-boosted PDT + Sorafenib	Experimental tumor models	GSH depletion, MDA accumulation, ferroptosis enhancement	Improved antitumor efficacy and lymphocyte infiltration	[151]
Preclinical	Combination therapy	Multiple experimental cancer models	Ferroptosis inducers combined with immune checkpoint blockade	Promising synergistic strategy in preclinical studies	[152]
Preclinical	Iron oxide nanocluster nanoparticles	Experimental tumor models	Fenton reaction, iron release, immune activation	Proof-of-concept nanoplatform	[153]
Preclinical	NO donors	Experimental macrophage models	Regulation of macrophage ferroptosis sensitivity	Mechanistic evidence from preclinical studies	[154]

**Note 1:** Therapeutic strategies are classified according to the highest level of available evidence at the time of manuscript preparation.

**Note 2:** FDA approval refers to the approved clinical indication of the drug and does not necessarily indicate regulatory approval specifically for ferroptosis-targeted cancer therapy.

**Note 3:** Several therapies remain under preclinical investigation despite demonstrating promising ferroptosis-inducing activity in experimental models.

### 4.3 Nanoparticles that induce ferroptosis

Certain nanoparticles induce ferroptosis by disrupting intracellular iron homeostasis and promoting excessive reactive oxygen species (ROS) generation. Representative ferroptosis-inducing nanoplatforms and their mechanisms of action are summarized in Table 4. For example, C′ dots functionalized with surface-bound iron sensitize amino acid-deprived cancer cells by delivering bioavailable iron through silicon-based nanoparticles [155]. UPDA-PEG@Fe^2+^/^3+^ nanoparticles similarly regulate intracellular iron release to enhance ferroptotic responses [156]. Superparamagnetic iron oxide nanoparticles (SPIONs) increase intracellular iron levels through particle degradation [157], thereby promoting ferroptosis and, in some contexts, apoptosis, necrosis, and autophagy [158]. In addition, tungsten sulfide (WS_2_) and molybdenum disulfide (MoS_2_) nanosheets induce lysosomal disruption and iron release [159], whereas GA-Fe(II) nanoparticles promote ferroptosis by accelerating Fenton chemistry and ROS production [160].

**Table 4:** Application of nanoparticles for ferroptotic cancer therapy.

Regulators	Application	Targets	Mechanisms	References
DAR	antitumor drug delivery	Human MCF7 breast cancer Mouse mammary 4T1 tumor	Doxorubicin	[161]
ACC@DOX–CaSi–PAMAM–FA/mPEG	antitumor drug delivery	Mouse mammary 4T1 tumor Human A375 melanoma	Doxorubicin	[162]
DGU:Fe	antitumor drug delivery	Human MCF7 breast cancer Mouse mammary 4T1 tumor	Doxorubicin	[163]
PtH@FeP	antitumor drug delivery	Human HepG2 hepatocellular carcinoma Human A2780 ovarian ca	Cisplatin	[164]
FeGd-HN@Pt@LF/RGD2	antitumor drug delivery	Human U-87 MG glioblastoma	Cisplatin	[161]
UCNP@LP(Azo-CA4)	antitumor drug delivery	Human MDA-MB-231 breast cancer	Azobenzene combretastatin A4	[161]
Tf-LipoMof@PL	tumor targeting	Mouse mammary 4T1 tumor	Trasferrin	[165]
Malt-PEG-Abz@RSL3	tumor targeting	Human HepG2 hepatocellular carcinoma	GLUT	[166]
NMIL-100@GOx@C	tumor targeting	Mouse mammary 4T1 tumor	Starvation and glucose oxidase	[167]
AMSNs	tumor targeting	Human Huh 7 hepatocellular carcinoma	Arginine transporter	[161]
API-Fe2O3/Fe3O4@mSiO2-HA	tumor targeting	Human A549 lung carcinoma	Hyaluronic acid and magnetic field	[168]
mPEG-Azo-PBLA@RSL3	tumor targeting	Mouse mammary 4T1 tumor	Hypoxia	[161]
FaPEG-MnMSN@SFB	tumor targeting	Human HepG2 hepatocellular carcinoma Mouse mammary 4T1 tumor Human A549 lung carcinoma	Hyperoxidative condition	[161]
RSL3@COF-Fc	tumor targeting	Human HT1080 fibrosarcoma Human MCF7 breast cancer Human HCT116 colon carcinoma	Hyperoxidative condition	[169]
CSO-SS-Cy7-Hex/SPION/Srfn	tumor targeting	Mouse mammary 4T1 tumor Human MDA-MB-231 breast cancer	Hyperoxidative condition	[170]
TA-Fe/ART@ZIF	tumor targeting	Human MDA-MB-231 breast cancer	pH responsive	[171]
HA@MOF	tumor targeting	Mouse mammary 4T1 tumor	pH responsive	[172]
WA-NPs	tumor targeting	Human IMR-32 neuroblastoma	pH responsive	[173]
Pyrite-peroxidase nanozyme	tumor targeting	Mouse CT26 colon adenocarcinoma	Folate/folate receptor	[161]
PSAF NCs	tumor targeting	Mouse mammary 4T1 tumor	pH responsive	[161]
MIL88B/RSL3	immunomodulation	Mouse mammary 4T1 tumor	M1 polarization of TAMs	[161]
ZVI-NP	immunomodulation	Human A549 lung carcinoma Human H460 lung carcinoma Human HT1299 lung carcinoma Mouse LLC lung carcinoma	M1 polarization of TAMs CD8+T cell increase Regulatory T cells reduction	[174]
Pa-M/Ti-NC	immunomodulation	Mouse mammary 4T1 tumor	M1 polarization of TAMs Generation of an immunogenic microenvironment that favors ferroptosis	[175]
HLCaP	radiofrequency ablation, hyperthermia, and photodynamic therapy	Mouse mammary 4T1 tumor Mouse H22 hepatocellular carcinoma Rabbit VX2 hepatocellular carcinoma	Radiofrequency ablation	[161]
GBP@Fe3O4	radiofrequency ablation, hyperthermia, and photodynamic therapy	Human MBA-MD-231 breast cancer Human 786-O renal cell carcinoma Human PC3 prostate cancer Human C4-2 prostate cancer	Hyperthermia	[161]
Fe3O4-PLGA-Ce6	radiofrequency ablation, hyperthermia, and photodynamic therapy	Mouse mammary 4T1 tumor	Photodynamic therapy	[176]
PEG-Fns	radiofrequency ablation, hyperthermia, and photodynamic therapy	Mouse mammary 4T1 tumor	Photodynamic therapy	[177]
PB@FePt-HA-g-PEG	radiofrequency ablation, hyperthermia, and photodynamic therapy	Mouse mammary 4T1 tumor Human MCF7 breast cancer	Photodynamic therapy	[178]
SRF@Hb-Ce6	radiofrequency ablation, hyperthermia, and photodynamic therapy	Mouse mammary 4T1 tumor Human A549 lung carcinoma Human HepG2 hepatocellular carcinoma	Photodynamic therapy	[179]
Ce6@RMOF	radiofrequency ablation, hyperthermia, and photodynamic therapy	Mouse mammary 4T1 tumor	Photodynamic therapy	[161]
SRF@Fe^III^TA	radiofrequency ablation, hyperthermia, and photodynamic therapy	Mouse mammary 4T1 tumor	Photodynamic therapy	[161]

Some NPs boost ROS via GSH depletion/Fenton-like processes. LDL-DHA CPMNS block GSH and boost lipid ROS [180]. ChA CQDs oxidize GSH, disrupting repair [181]. Co^2+^ oxidation depletes GSH [182]; Mo(V) to Mo(VI) conversion induces ferroptosis [183]. ZnO NPs release lethal Zn^2+^ [184]. Photosynthetic microcapsules generate ROS via cyanobacteria O_2_ [185].

Combining NPs with small molecules/drugs enhances effects. Targeted NPs deliver inducers [186]. Acidity-activatable RSL3 NPs trigger ferroptosis [187]. Ultrasound-NPs combine for efficacy [188]. Table 5 provides a direct comparison between small-molecule ferroptosis inducers and nanoparticle-based systems in terms of mechanism, pharmacokinetics, tumor targeting, safety, and clinical status.

**Table 5:** Small molecule vs. nanoparticle inducers.

Feature	Small-molecule -inducers	Nanoparticle-based inducers	References
Mechanism	Direct inhibition of GPX4, SLC7A11, or FSP1	Fenton reaction, GSH depletion, lysosomal iron release, photothermal enhancement	[189]
Pharmacokinetics	Often poor solubility, rapid clearance, off-target toxicity	Potentially improved circulation and controlled release; pharmacokinetics are highly dependent on particle composition and may be limited by heterogeneous biodistribution and uptake by the mononuclear phagocyte system	[190]
Tumor specificity	Low (systemic exposure)	Potentially improved through passive and active targeting, but tumor accumulation is heterogeneous and substantial off-target organ uptake may occur	[190]
Safety concerns	Hepatotoxicity, neurotoxicity	Accumulation in RES organs (liver, spleen), long-term iron overload	[191]
Clinical status	Sorafenib (FDA-approved for HCC/RCC); erastin/RSL3 preclinical	Mostly preclinical; a few in Phase I (e.g., iron oxide nanoparticles)	[192]
Combination potential	Limited by toxicity	Excellent – can co-deliver drugs, photosensitizers, or immunomodulators	[193]
Clearance and persistence	Often dependent on molecular metabolism and renal/hepatic elimination	Highly dependent on size, composition, biodegradability, and surface properties; persistent materials may accumulate in tissues	[194]

### 4.4 Translational considerations for ferroptosis-inducing nanoparticles

Despite remarkable progress in the development of ferroptosis-inducing nanoplatforms, several translational challenges must be addressed before their widespread clinical application becomes feasible [195]. One of the foremost considerations is biosafety, as nanomaterials may induce unintended oxidative stress, inflammatory responses, complement activation, or accumulation in non-target organs, particularly the liver, spleen, and kidneys [196]. Long-term toxicity and potential immunogenicity therefore require comprehensive evaluation using standardized preclinical models [197].

Another critical consideration is biodegradability. Biodegradable nanocarriers composed of lipids, polymers, proteins, or other biocompatible materials generally demonstrate more favorable safety profiles than persistent inorganic nanoparticles, which may remain in tissues following repeated administration [198,199]. Rational material selection and surface engineering are therefore essential for minimizing long-term toxicity while maintaining therapeutic efficacy.

Biodistribution represents an equally important determinant of translational performance. The in vivo distribution of nanoparticles is influenced by particle size, shape, surface charge, composition, protein-corona formation, circulation time, and interactions with the mononuclear phagocyte system [200]. Consequently, systemic administration does not necessarily result in preferential tumor accumulation, and substantial uptake by the liver and spleen may limit the effective tumor dose while increasing the risk of off-target toxicity. Passive accumulation through the enhanced permeability and retention effect is also heterogeneous among tumors and should not be considered a universally reliable mechanism of tumor targeting. Active targeting strategies, including ligand modification and tumor-responsive delivery systems, may improve cellular selectivity but do not eliminate the need for comprehensive in vivo biodistribution studies [201].

Clearance and persistence should likewise be considered during nanoplatform design. The elimination pathway depends on the physicochemical characteristics and biodegradability of the nanomaterial and may involve renal excretion, hepatobiliary elimination, metabolic degradation, or prolonged retention within tissues. Persistent or slowly degradable materials may produce cumulative toxicity following repeated administration, whereas premature degradation may cause uncontrolled release of iron or other ferroptosis-promoting components. Therefore, the kinetics of nanoparticle degradation, cargo release, organ retention, and systemic clearance should be evaluated together rather than considered independently [202,190].

Biocompatibility is particularly important for ferroptosis-inducing nanotherapeutics because their intended mechanism involves the generation of oxidative stress or perturbation of cellular redox homeostasis [203]. A therapeutic window must therefore be established in which ferroptotic stress is preferentially induced within tumor tissue without causing clinically significant injury to normal cells or organs. Particular attention should be given to hepatic, renal, hematological, immunological, and neurological toxicity, as well as the possibility of unintended ferroptosis in non-malignant tissues. Comprehensive pharmacokinetic, biodistribution, biodegradation, and long-term safety studies will therefore be essential before promising ferroptosis-inducing nanoplatforms can advance toward clinical application [190].

The pharmacokinetic behavior of ferroptosis-inducing nanoparticles also plays a pivotal role in determining therapeutic outcomes [204]. Nanoparticle size, surface charge, morphology, protein corona formation, and circulation half-life collectively influence biodistribution, tumor accumulation, and systemic clearance [205]. Strategies such as PEGylation, biomimetic membrane coating, and stimuli-responsive nanocarriers have been developed to prolong circulation time while reducing premature clearance by the mononuclear phagocyte system [206].

Furthermore, efficient tumor targeting remains a major determinant of clinical success [207]. Passive accumulation through the enhanced permeability and retention (EPR) effect alone is often insufficient because of interpatient variability and tumor heterogeneity [208]. Consequently, active targeting approaches utilizing antibodies, peptides, aptamers, or ligand-receptor interactions are increasingly being investigated to improve tumor selectivity and therapeutic efficacy while minimizing off-target toxicity [209].

Collectively, future clinical translation of ferroptosis-based nanomedicine will depend not only on maximizing ferroptosis induction but also on optimizing biosafety, biodegradability, pharmacokinetic properties, manufacturing reproducibility, and regulatory compliance. Continued interdisciplinary collaboration among materials scientists, pharmacologists, oncologists, and regulatory agencies will be essential for advancing these promising therapeutic platforms toward clinical implementation [204].

### 4.5 Nucleic acids and proteins with stimulatory effects

Non-coding RNAs like miRNAs regulate ferroptosis [210]. Post-SCI, miR-672-3p upregulates, blocking FSP1 and inducing ferroptosis [211]. NEAT1 promotes ferroptosis via miR-362-3p antagonism and MIOX activation, though other NEAT1 transcripts inhibit ACSL4 [212].

IFN-γ from CD8^+^ T cells downregulates Xc^−^ subunits [213]. SLC7A11 is suppressed by p53, BAP1, and miR-672-3p [214]. TCA cycle links to glutaminolysis; deleting ACC/fumarase/α-KGDH prevents cysteine-deficiency ferroptosis [12]. PKCβII phosphorylates ACSL4, increasing LPO [188]. NCOA4 degrades ferritin lysosomally [45].

## 5 Determinants of ferroptosis response in cancer

### 5.1 Tumor heterogeneity and ferroptosis sensitivity

Intratumoral heterogeneity is a major barrier to effective ferroptosis-based therapy. Single-cell RNA sequencing has revealed that within the same tumor, subpopulations of cancer cells display widely divergent expression of ferroptosis core regulators [215]. For example, SLC7A11 (the light chain of system Xc^−^) is expressed in a mosaic pattern, with some cells exhibiting high levels and others being essentially negative [216]. Similarly, ACSL4 expression, which dictates sensitivity by enriching membranes with pro-ferroptotic polyunsaturated fatty acids, varies considerably. Cells with low ACSL4 are intrinsically resistant [217]. Moreover, mutational heterogeneity (e.g., p53 wild-type versus mutant, KRAS G12C versus other alleles) can either sensitize or protect [218]. To overcome this, combination strategies targeting multiple nodes of the ferroptosis pathway (e.g., simultaneous inhibition of GPX4 and FSP1) are likely required, rather than relying on a single agent [219].

### 5.2 The tumor microenvironment: iron, immunity, and ferroptosis

The tumor microenvironment (TME) profoundly influences ferroptosis susceptibility. Cancer-associated fibroblasts (CAFs) secrete exosomes enriched in miR-522, which directly suppresses ALOX15 expression in cancer cells, thereby reducing lipid peroxidation and conferring ferroptosis resistance [112]. Tumor-associated macrophages (TAMs) display a spectrum of phenotypes: M2-polarized TAMs typically express high levels of GPX4 and are resistant to ferroptosis, whereas M1-polarized TAMs have low GPX4 and are highly sensitive. This differential sensitivity can be exploited to repolarize TAMs from a pro-tumor M2 to an anti-tumor M1 state using ferroptosis inducers [220]. Furthermore, CD8^+^ T cells infiltrating the TME produce interferon-γ (IFN-γ), which downregulates SLC7A11 expression in cancer cells, making them more vulnerable to ferroptosis. Thus, immunotherapies that increase CD8^+^ T cell infiltration (e.g., anti-PD-1) may synergize with ferroptosis inducers [213,221].

### 5.3 Determinants of differential ferroptosis sensitivity across cancers

The basal sensitivity to ferroptosis varies markedly among cancer types. Factors driving this variability include: (1) Basal GPX4 and SLC7A11 levels – pancreatic and lung adenocarcinomas often exhibit high expression, conferring resistance, while some lymphomas and leukemias have low levels and are highly sensitive [222]. (2) Lipid metabolism profile – cancers with high ACSL4 expression (e.g., triple-negative breast cancer, clear cell renal carcinoma) are primed for ferroptosis, whereas those relying on monounsaturated fatty acids (e.g., prostate cancer) are resistant [223]. (3) Iron handling – ferritin heavy chain (FTH1) overexpression reduces labile iron and protects against ferroptosis; conversely, mutations that elevate TfR1 increase sensitivity [224]. (4) Mutation status – p53 loss-of-function can impair ferroptosis by reducing PUFA synthesis, while KRASG12D mutations sensitize cells to GPX4 inhibition [225]. These determinants should guide patient selection for ferroptosis-targeted therapies.

### 5.4 Clinically relevant biomarkers for predicting ferroptosis sensitivity

Ferroptosis sensitivity is determined by the balance between iron availability, phospholipid composition, lipid-peroxidation burden, and cellular antioxidant defenses. Accordingly, biomarkers that reflect these processes may facilitate patient stratification, prediction of therapeutic response, pharmacodynamic monitoring, and identification of resistance mechanisms. However, an important distinction should be made between mechanistically informative ferroptosis-related markers and clinically validated predictive biomarkers. Although numerous ferroptosis-associated genes and metabolites have demonstrated prognostic or predictive value in retrospective and preclinical studies, no single biomarker has yet been established as a universally validated companion diagnostic for ferroptosis-targeted cancer therapy. Recent work has consequently shifted toward multimarker and multi-omics approaches that capture the dynamic and context-dependent nature of ferroptosis susceptibility [226]. The principal candidate biomarkers, their biological rationale, potential clinical applications, and current limitations are summarized in Table 6.

**Table 6:** Candidate biomarkers for predicting ferroptosis sensitivity and monitoring ferroptosis-targeted therapy.

Biomarker class	Representative biomarker	Biological rationale	Potential clinical application	Major limitation	References
Antioxidant defense	GPX4	Detoxifies phospholipid hydroperoxides	Predict dependence on GPX4-mediated defense; monitor target engagement	Expression does not necessarily reflect enzymatic activity	[90]
Cystine/GSH metabolism	SLC7A11	Controls cystine uptake and supports GSH synthesis	Patient stratification for System Xc^−^-targeted approaches	Metabolic compensation can occur	[6]
Lipid remodeling	ACSL4	Enriches PUFA-containing phospholipids susceptible to peroxidation	Identify ferroptosis-prone tumors	Context-dependent and influenced by lipid composition	[227]
GPX4-independent defense	FSP1	Maintains CoQ10-dependent suppression of lipid radicals	Identify resistance to GPX4-directed therapy	Clinical predictive value remains unvalidated	[228]
Lipid peroxidation	4-HNE, MDA	Reflect lipid oxidation	Pharmacodynamic monitoring	Not specific to ferroptosis	[229]
Oxidized phospholipids/lipid ROS	OxPLs, lipid-ROS signatures	Directly reflects lipid oxidation	Pharmacodynamic assessment and research-based patient stratification	Assay standardization and clinical accessibility	[226]
Iron metabolism	TFRC, ferritin/labile iron parameters	Determines iron availability for ROS generation	Complementary assessment of ferroptosis susceptibility	Total iron/ferritin does not directly represent labile iron	[230]
Composite molecular signatures	Multi-gene/omics ferroptosis signatures	Integrates multiple determinants of sensitivity	Potential precision-oncology stratification	Requires prospective clinical validation	[226]

GPX4 is one of the most biologically informative candidate biomarkers because it represents a central defense against phospholipid hydroperoxidation. High GPX4 expression or activity may indicate strong ferroptosis-protective capacity and, in appropriate biological contexts, increased dependence on GPX4 for survival. Conversely, tumors with impaired GPX4 activity may exhibit greater susceptibility to further perturbation of lipid peroxide detoxification. However, GPX4 abundance alone should not be interpreted as a direct surrogate for ferroptosis sensitivity because compensatory GPX4-independent pathways, particularly FSP1/CoQ10, GCH1/BH4, and mitochondrial DHODH systems, can substantially modify the cellular response. Importantly, recent studies have shown that post-translational regulation of GPX4 can also influence ferroptosis sensitivity, indicating that measurements of protein abundance may not fully capture functional GPX4 activity [231].

SLC7A11 provides another potentially informative marker because it controls cystine import through system Xc^−^ and thereby supports intracellular cysteine availability, glutathione synthesis, and GPX4-dependent antioxidant defense. High SLC7A11 expression may therefore identify tumors with enhanced dependence on the cystine–GSH–GPX4 axis and potential sensitivity to interventions that disrupt cystine uptake or glutathione metabolism. Nevertheless, SLC7A11 expression should be interpreted together with GPX4, GSH availability, transsulfuration capacity, and other antioxidant pathways because metabolic compensation can preserve redox homeostasis despite inhibition of system Xc^−^. The existing manuscript already notes that dysregulation of the SLC7A11–GSH–GPX4 axis is associated with ferroptosis sensitivity; the present subsection extends this observation by placing SLC7A11 within a patient-stratification framework [232].

ACSL4 is particularly relevant as a marker of the lipid substrate landscape required for ferroptosis. ACSL4 promotes incorporation of polyunsaturated fatty acids into phospholipids, thereby increasing the pool of membrane lipids that can undergo peroxidation. High ACSL4 expression has consequently been associated with ferroptosis-prone phenotypes in several experimental cancer models, whereas reduced ACSL4 activity can confer resistance. However, ACSL4 should not be considered an isolated predictor because the abundance and composition of polyunsaturated phospholipids also depend on LPCAT3, lipoxygenases, fatty-acid availability, and competing lipid-remodeling pathways. The existing discussion of ACSL4 and intertumoral differences therefore provides a strong mechanistic foundation for considering ACSL4 as part of a biomarker panel rather than as a standalone clinical test [233].

FSP1 is particularly important for identifying tumors in which GPX4-independent antioxidant defense may limit therapeutic efficacy. By supporting the reduction of coenzyme Q10 and suppressing lipid-radical propagation, FSP1 provides an alternative defense against ferroptosis. Consequently, tumors with high FSP1 activity may display relative resistance to GPX4-directed approaches, whereas combined targeting of GPX4-and FSP1-dependent defenses may overcome this resistance in selected contexts. Recent experimental studies further emphasize that FSP1 activity can vary among tumor types, reinforcing the concept that GPX4 and FSP1 should be evaluated as complementary rather than interchangeable biomarkers [234].

Lipid-peroxidation products may provide a complementary class of pharmacodynamic biomarkers because they more directly reflect the biochemical event underlying ferroptotic cell death. Malondialdehyde (MDA) and 4-hydroxynonenal (4-HNE) are products of lipid-peroxidation reactions and can be detected using biochemical, immunohistochemical, or other analytical approaches. The manuscript already identifies MDA and 4-HNE as products generated during lipid-peroxidation processes [75,76]. Recent work has also highlighted 4-HNE as a biologically active lipid-derived electrophile rather than merely a passive marker of oxidative damage. Therefore, increased MDA or 4-HNE may support evidence of enhanced lipid peroxidation, but these measurements should not be interpreted as specific proof of ferroptosis because lipid peroxidation and reactive aldehyde generation can occur in other forms of oxidative stress and regulated cell death [235].

Direct measurement of lipid reactive oxygen species and oxidized phospholipids may provide greater mechanistic specificity. Fluorescent probes such as C11-BODIPY can detect changes in lipid oxidation in experimental systems, whereas lipidomic analyses can characterize oxidized phospholipid species more comprehensively. Such approaches are particularly valuable for pharmacodynamic studies because ferroptosis is a dynamic process and static measurement of a single protein may not accurately reflect the current ferroptotic state. However, many of these assays remain primarily research tools and require standardization before routine clinical application. Recent reviews similarly emphasize the potential value of lipid-peroxidation products and ferroptosis-related molecular markers while highlighting the challenges associated with transient biomarkers and their clinical detectability [236].

Iron-metabolism markers may provide an additional dimension of biomarker assessment. Transferrin receptor 1 (TFRC/TfR1), ferritin, the labile iron pool, and regulators of ferritinophagy may influence ferroptosis susceptibility by controlling the availability of redox-active iron. However, total tissue iron or serum ferritin should not be equated directly with ferroptotic activity because iron is distributed among multiple intracellular pools with distinct biological functions. Consequently, iron-related biomarkers are likely to be most informative when combined with markers of lipid peroxidation and antioxidant capacity rather than used independently [237].

Taken together, the current evidence supports a multidimensional biomarker framework rather than reliance on a single ferroptosis marker (Table 6). A clinically informative panel could integrate antioxidant defenses (GPX4, SLC7A11, and FSP1), lipid susceptibility (ACSL4 and related lipid-remodeling pathways), iron availability (TFRC and ferritin-related parameters), and biochemical evidence of lipid peroxidation (4-HNE, MDA, oxidized phospholipids, and lipid-ROS signatures). Such multimarker strategies may be further refined using transcriptomics, proteomics, lipidomics, and spatial or single-cell analyses to capture intratumoral heterogeneity. Nevertheless, the field currently lacks standardized thresholds, harmonized assays, and prospective clinical validation demonstrating that any individual ferroptosis biomarker or composite signature can reliably predict benefit from a ferroptosis-targeted therapy. Therefore, biomarker development should proceed alongside clinical trials, with prospective validation of predictive performance, reproducibility, and clinical utility before implementation in precision oncology [238].

In practical terms, the principal candidate biomarkers represent complementary dimensions of ferroptosis biology. GPX4 reflects lipid-peroxide detoxification capacity, but expression does not necessarily represent functional enzymatic dependence. SLC7A11 indicates the activity of cystine uptake and glutathione metabolism, although metabolic compensation may preserve antioxidant defenses. ACSL4 reflects the availability of peroxidation-prone phospholipid substrates but is strongly influenced by the broader lipid-metabolic context. FSP1 provides information regarding GPX4-independent antioxidant protection and potential resistance to GPX4-directed approaches, although its clinical predictive value remains unvalidated. Finally, iron-related parameters, including TFRC and ferritin-associated measures, provide complementary information regarding iron availability but do not directly quantify the labile iron pool or ferroptotic activity. These limitations support the use of integrated biomarker panels rather than reliance on a single molecular marker [239].

## 6 Inhibitors of ferroptosis

Inhibitors are mostly small molecules, aiding tumor treatment, stroke, and immunotherapy [240]. Table 2 summarized inhibitors that block ferroptosis by targeting key pathways like iron metabolism, lipid peroxidation, and the GSH/GPX4 axis.

### 6.1 Small-molecule inhibitors reducing iron levels

Iron chelators like cyclopirox olamine (CPX) degrade ferritin [241]. Deferoxamine (DFO) blocks ferroptosis post-SCI [242]. Deferiprone (DFP) and deferasirox (DFX) chelate iron [243].

### 6.2 Small-molecule inhibitors reducing lipid peroxides

Ferrostatin-1 (Fer-1) prevents LPO via its amine [60]; rescues p53-induced ferroptosis [244] and boosts GSH in oligodendrocytes [112]. Liproxstatin-1 and vitamin E block erastin/GPX4 loss [245]. Analogs (SRS15-72 B/A, SRS16-80/86) vary in efficacy [246]. FSP1 converts vitamin K to VKH_2_, preventing LPO [247].

### 6.3 Small-molecule inhibitors affecting the gsh/gpx4 axis

β-Mercaptoethanol (-ME) suppresses Xc^−^, enabling cysteine uptake [248]. Selenium upregulates GPX4 via TFAP2c/Sp1 [240]. Bardoxolone (CDDO) blocks HSP90, preventing GPX4 degradation [110].

### 6.4 Nucleic acids and proteins with inhibitory effects

Prominin-2 stimulates ferritin secretion via exosomes [249]. LINC00336 inhibits RNA polymerase II [250]. RP11-89 sponges miR-129-5p [186]. miR-522 suppresses ALOX15 [112].

In a study using DOX/TA/IR820 (DAR) nanoparticles, Xiong et al. used DOX/TA/IR820 NPs [251]. DAR accumulates via ferritinophagy, disrupting lysosomes and iron sequestration. Laser (808 nm) boosts release and ROS in MCF7 cells, inducing ER stress and ICD. In vivo, DAR/laser synergizes in MCF7/4T1 tumors [161].

ACC1 controls ferroptosis via LKB1-AMPK; AMPK phosphorylates/inhibits ACC1 [252,253]. GPX4-deficient M1 TAMs are ferroptosis-susceptible.

## 7 Current limitations and future perspectives of ferroptosis-based cancer therapy

Although ferroptosis has emerged as a highly promising therapeutic strategy for cancer treatment, several biological, pharmacological, and translational challenges continue to impede its successful clinical implementation. While numerous preclinical studies have demonstrated potent antitumor activity through ferroptosis induction, relatively few approaches have advanced into clinical evaluation. These limitations arise from the complex interplay between tumor biology, drug pharmacology, the tumor microenvironment, and patient-specific heterogeneity [254]. A comprehensive understanding of these barriers is therefore essential for developing safe, effective, and clinically translatable ferroptosis-based therapies.

### 7.1 Tumor heterogeneity and variable ferroptosis susceptibility

One of the greatest obstacles to ferroptosis-based therapy is the remarkable heterogeneity that exists both between different tumor types and within individual tumors [255]. Cancer cells exhibit substantial differences in iron metabolism, antioxidant capacity, lipid composition, metabolic reprogramming, and expression of key ferroptosis regulators, including GPX4, SLC7A11, ACSL4, FSP1, NRF2, and ferritin [30]. Consequently, tumors with apparently similar histological characteristics may respond very differently to ferroptosis-inducing agents [256]. Intratumoral heterogeneity further complicates treatment because resistant subpopulations can survive therapy and drive disease recurrence [257]. These observations emphasize the need for biomarker-guided patient stratification and personalized therapeutic approaches rather than uniform treatment strategies [255].

### 7.2 Off-target toxicity and normal tissue injury

Because ferroptosis is fundamentally driven by iron-dependent lipid peroxidation, normal tissues with high oxidative metabolism may also be vulnerable to ferroptotic injury [258]. Excessive ferroptosis has been implicated in neurodegenerative disorders, ischemia-reperfusion injury, acute kidney injury, and liver damage, highlighting the potential risk of systemic ferroptosis induction [259]. Achieving selective tumor cell killing while preserving normal tissues therefore remains a central challenge [228]. Future therapeutic strategies should incorporate tumor-specific drug delivery systems, targeted nanoparticles, antibody-drug conjugates, or ligand-mediated targeting approaches to maximize therapeutic selectivity and minimize off-target toxicity [260].

### 7.3 Drug delivery and pharmacokinetic limitations

Many ferroptosis-inducing compounds exhibit suboptimal pharmacokinetic properties, including poor aqueous solubility, rapid systemic clearance, limited tumor penetration, and inadequate intracellular accumulation [261]. Furthermore, biological barriers such as abnormal tumor vasculature, elevated interstitial fluid pressure, and extracellular matrix remodeling can significantly reduce drug delivery efficiency [262]. Although nanotechnology-based delivery systems have demonstrated considerable promise in overcoming these limitations, challenges related to large-scale manufacturing, reproducibility, long-term biosafety, biodegradability, and regulatory approval remain unresolved [263]. Optimizing drug formulation and delivery platforms will therefore be essential for successful clinical translation [264].

### 7.4 Adaptive resistance mechanisms

Cancer cells possess remarkable metabolic plasticity that enables them to adapt to ferroptotic stress [265]. Upregulation of antioxidant pathways involving GPX4, FSP1, NRF2, DHODH, and the GCH1–BH4 axis can substantially reduce lipid peroxidation and promote therapeutic resistance [18]. Alterations in iron homeostasis, ferritin metabolism, lipid remodeling, and cystine uptake may further diminish ferroptosis sensitivity. These adaptive responses indicate that monotherapy targeting a single ferroptosis pathway may provide only transient clinical benefit. Rational combination therapies targeting multiple ferroptosis-regulatory mechanisms simultaneously are therefore likely to achieve more durable therapeutic responses [14].

### 7.5 Influence of the tumor microenvironment

The tumor microenvironment exerts profound effects on ferroptosis sensitivity through interactions among cancer cells, immune cells, cancer-associated fibroblasts, endothelial cells, and extracellular matrix components. Factors such as hypoxia, nutrient availability, cytokine signaling, and immune-cell infiltration can either promote or suppress ferroptosis depending on the biological context. Moreover, macrophage polarization and T-cell activation influence iron metabolism and oxidative stress, thereby affecting therapeutic efficacy. A more comprehensive understanding of these complex cellular interactions will facilitate the rational design of combination therapies integrating ferroptosis induction with immunotherapy, chemotherapy, radiotherapy, or targeted molecular agents [14].

### 7.6 Clinical translation and future directions

Despite substantial advances in mechanistic understanding, relatively few ferroptosis-targeted therapies have progressed into clinical development [266]. Future progress will require standardized biomarkers for patient selection, robust pharmacodynamic assays for monitoring treatment response, improved preclinical models that more accurately reflect human tumor biology, and well-designed clinical trials evaluating safety, efficacy, and optimal combination regimens [267]. Advances in artificial intelligence, multi-omics technologies, precision oncology, and nanomedicine are expected to facilitate patient stratification and accelerate the clinical translation of ferroptosis-based therapies [268]. Ultimately, integrating molecular biomarkers with rational combination strategies and innovative drug-delivery platforms may enable ferroptosis induction to become a clinically effective component of personalized cancer therapy [190].

## 8 Conclusion

This review has synthesized the regulatory mechanisms of ferroptosis with a focused lens on cancer therapy. Key hallmarks include iron-dependent ROS accumulation via the Fenton reaction and GPX4 inactivation, with the Xc^−^/GSH/GPX4 and FSP1/CoQ10 axes offering prime therapeutic targets. We have highlighted emerging strategies beyond conventional small molecules and nanoparticles, such as cold atmospheric plasma, which physically induces ferroptosis through ROS generation and GPX4 depletion. Tumor heterogeneity, the microenvironment, and differential sensitivity among cancer types must be considered to design effective combination regimens. Finally, we have identified critical limitations of current preclinical models and advocate for more physiologically relevant systems (PDOs, GEMMs, humanized mice) to accelerate clinical translation. Future work should focus on biomarker-driven patient selection and rationally designed combination therapies that leverage ferroptosis together with immunotherapy, radiotherapy, or chemotherapy.

Most preclinical studies on ferroptosis inducers rely on conventional 2D cell cultures or subcutaneous xenografts in immunodeficient mice. These models poorly recapitulate the human tumor microenvironment, particularly the immune component and the complex iron distribution within tumors. For instance, the activity of CD8^+^ T cells, which modulate ferroptosis via IFN-γ, is absent in xenografts. Moreover, standard cell culture media contain supraphysiological concentrations of cysteine and iron, which can blunt ferroptosis sensitivity. To improve clinical predictivity, we recommend using: (i) patient-derived organoids (PDOs) maintained in defined media that mimic the human tumor nutrient profile; (ii) autochthonous genetically engineered mouse models (GEMMs) with intact immune systems; and (iii) humanized mouse models reconstituted with patient immune cells. Such models will better capture the heterogeneity and dynamic nature of ferroptosis responses in patients.

## CRediT authorship contribution statement

**Mansoor Khaledi:** Supervision, Project administration. **Hasti Ahani:** Investigation, Formal analysis, Data curation. **Faezeh Rezaei Tohid:** Methodology, Investigation. **Fatemeh Kazemi:** Methodology, Formal analysis, Data curation. **Elham Amirkhani:** Formal analysis, Data curation. **Zeynab Daliri:** Methodology, Investigation. **Sahar Yadegari:** Methodology, Data curation. **Kosar Moarefvand:** Investigation, Formal analysis. **Behnam Poureslamfar:** Visualization, Validation. **Fatemeh Sameni:** Writing – review & editing, Writing – original draft. **Sajjad Yazdansetad:** Supervision.

## Clinical trial number

not applicable.

## Funding

The author(s) received no financial support for the research and authorship of this article.

## Declaration of competing interest

I have nothing to declare.

## Data Availability

No data was used for the research described in the article.
