# Supplementary material for: Targeting ferroptosis in cancer: molecular mechanisms and emerging therapeutic strategies
Source: Biochem Biophys Rep. 2026 Oct 9;48:102825. doi: 10.1016/j.bbrep.2026.102825 (PMC13677573; doi:10.1016/j.bbrep.2026.102825)
Supplement: Supplementary file 1 [file mmc1.docx]

**Supplementary Table S1 – Ferroptosis in non‑cancer pathologies**

| **Disease category** | **Examples** | **References** |
| --- | --- | --- |
| Neurodegenerative | Alzheimer’s, Parkinson’s, Huntington’s | [269-271] |
| Ischemic injury | Myocardial infarction, stroke, kidney I/R | [272, 273] |
| Liver diseases | Alcoholic / non‑alcoholic fatty liver disease | [272, 274] |
| Others | Traumatic brain injury, multiple sclerosis | [270, 271] |
